# Acute inorganic nitrate intake increases regional insulin action in the brain: Results of a double-blind, randomized, controlled cross-over trial with abdominally obese men

**DOI:** 10.1016/j.nicl.2022.103115

**Published:** 2022-07-14

**Authors:** Jordi P.D. Kleinloog, Ronald P. Mensink, Ellen T.H.C. Smeets, Dimo Ivanov, Peter J. Joris

**Affiliations:** a Department of Nutrition and Movement Sciences, NUTRIM School of Nutrition and Translational Research in Metabolism, Maastricht University, Maastricht, The Netherlands; b Department of Cognitive Neuroscience, Faculty of Psychology and Neuroscience, Maastricht University, Maastricht, The Netherlands

**Keywords:** Abdominally obese, Arterial spin labelling, Brain insulin action, Cerebral blood flow, Intranasal insulin spray, Inorganic nitrate

## Abstract

**Aims:**

Improving brain insulin sensitivity may be a promising approach in the prevention and treatment of metabolic and cognitive diseases. Our aim was to investigate acute effects of inorganic nitrate on regional cerebral blood flow (CBF) responses to intranasal insulin in abdominally obese men.

**Methods:**

Eighteen apparently healthy men, aged 18–60 years and with a waist circumference ≥ 102 cm, participated in a randomized, double-blind, placebo-controlled cross-over trial. The study consisted of two test days separated by at least one week. Men received in random order a drink providing 10 mmol (i.e., 625 mg nitrate) potassium nitrate or an isomolar placebo drink with potassium chloride. Brain insulin action was assessed 120–150 min after the drinks by quantifying acute effects of nasal insulin on regional CBF using arterial spin labeling Magnetic Resonance Imaging. Glucose and insulin concentrations were measured at regular intervals, while blood pressure was determined fasted and at 240 min.

**Results:**

Inorganic nitrate intake increased regional insulin action in five brain clusters. The two largest clusters were located in the right temporal lobe (ΔCBF: 7.0 ± 3.8 mL/100 g/min, volume: 5296 mm^3^, P < 0.001; and ΔCBF: 6.5 ± 4.3 mL/100 g/min, volume: 3592 mm^3^, P < 0.001), while two other cortical clusters were part of the right frontal (ΔCBF: 9.0 ± 6.0 mL/100 g/min, volume: 1096 mm^3^, P = 0.007) and the left parietal lobe (ΔCBF: 6.1 ± 4.3 mL/100 g/min, volume: 1024 mm^3^, P = 0.012). One subcortical cluster was located in the striatum (ΔCBF: 5.9 ± 3.2 mL/100 g/min, volume: 1792 mm^3^, P < 0.001). No effects of nitrate were observed on CBF before administration. Following nitrate intake, circulating nitrate plus nitrite concentrations increased over time (P = 0.003), but insulin and glucose concentrations and blood pressure did not change.

**Conclusion:**

Acute inorganic nitrate intake may improve regional brain insulin action in abdominally obese men. These regions are involved in the regulation of different metabolic and cognitive processes.

The trial was registered on January 6th, 2021 at ClinicalTrials.gov as NCT04700241.

## Highlights

• Brain insulin resistance is a characteristic of age-related diseases like dementia.
• Inorganic nitrate may prevent these diseases by improving (brain) vascular function.
• Regional cerebral blood flow responses to intranasal insulin were assessed.
• Acute inorganic nitrate consumption increased regional insulin action in the brain.
• Affected regions play a role in the regulation of metabolic and cognitive processes.

## 1 Introduction

Brain insulin resistance, which can be defined as the failure of brain cells to adequately respond to insulin, is a characteristic of type 2 diabetes and several other diseases, such as dementia (Arnold et al., 2018, Kullmann et al., 2016, Kullmann et al., 2020a). Therefore, improving brain insulin sensitivity by increasing the action of insulin in the brain may be a promising approach in the prevention and treatment of these age-related non-communicable diseases (Kullmann et al., 2022). Insulin may modulate cerebral blood flow (CBF) responses via direct vasodilatory effects (Hughes and Craft, 2016), while reduced brain insulin action was associated with cerebrovascular disturbances possibly via an impaired endothelium-dependent vasodilation (Akintola et al., 2017). Additionally, insulin regulates various metabolic and cognitive processes in the brain, and may also control food intake (Kullmann et al., 2020a). It has been shown that high brain insulin responsiveness prior to a two-year healthy lifestyle intervention resulted in more weight loss that was attributed to less visceral fat and less regain of fat mass during a nine-year follow-up (Kullmann et al., 2020b). However, trials investigating strategies that affect brain insulin action are still missing.

Inorganic nitrate, which is primarily found in beetroot and green leafy vegetables, may play a role in the prevention and comorbidities of insulin resistance and type 2 diabetes by improving nitric oxide (NO) homeostasis through the enterosalivary nitrate-nitrite-NO pathway (Bahadoran et al., 2015, Cordero-Herrera et al., 2020). Although inorganic nitrate is well-known for its beneficial effects on the peripheral vasculature (Bondonno et al., 2016), a limited number of studies have also already demonstrated an improved cerebrovascular function following the acute intake of nitrate (Joris et al., 2018). In fact, CBF, a sensitive marker for cerebrovascular function, improved after a diet providing 773 mg of dietary nitrate over a 24-hour feeding period in older participants (Presley et al., 2011). In addition, CBF acutely increased in young adults measured with transcranial doppler during exercise (Bond et al., 2013) and near-infrared spectroscopy during cognitive tasks (Wightman et al., 2015) after beetroot juice providing 750 mg and 342 mg of nitrate, respectively.

Effects of increased NO bioavailability after inorganic nitrate intake on regional CBF responses to intranasal insulin are unknown. Therefore, the aim of the present randomized, controlled, double-blind cross-over trial was to examine the acute effects of inorganic nitrate on brain insulin action. Focus was on abdominally obese men as they are known to have a reduced brain insulin responsiveness (Kullmann et al., 2020b). The action of insulin was assessed using a whole-brain approach by quantifying the acute effects of insulin as nasal spray on regional CBF (Kullmann et al., 2015, Kullmann et al., 2017) using the non-invasive perfusion method pseudo-continuous arterial spin labeling (ASL) magnetic resonance imaging (MRI) (Ivanov et al., 2017).

## 2 Methods

### 2.1 Study participants

Abdominally obese men were recruited by approaching participants from previous studies at Maastricht University, via online advertisements, and via local advertisements in university and hospital buildings. Men were invited for a screening visit if they were aged between 18 and 60 years and were right-handed. Additionally, participants had to meet the following criteria: minimal waist circumference 102 cm; no contra-indications for MRI imaging (e.g. any metallic implants or claustrophobia); stable body weight (weight gain or loss <3 kg in the past 3 months); non-smoker; no drug or alcohol abuse; no use of dietary supplements known to interfere with the main study outcomes; no diabetes; no use of medication known to affect blood pressure, lipid or glucose metabolism; no medical conditions that might interfere with the study (e.g. active cardiovascular disease); and no participation in another biomedical study within one month prior to the screening. During screening, blood pressure was measured in seated position according to the latest recommendations (Muntner et al., 2019). Systolic (SBP) and diastolic blood pressure (DBP) had to be lower than 160 mmHg and 100 mmHg, respectively. Additionally, a venous blood sample was drawn to determine if fasting plasma glucose was <7.0 mmol/L and fasting serum total cholesterol <8.0 mmol/L. Written informed consent was provided by all participants before screening. The study was conducted according to the guidelines described in the Declaration of Helsinki, approved by the Medical Ethics Committee of Maastricht University Medical Center (METC 20-078), and executed between January 2021 and May 2021. The trial was registered on January 6th 2021 at ClinicalTrials.gov as NCT04700241.

### 2.2 Study design

**Fig. 1:**
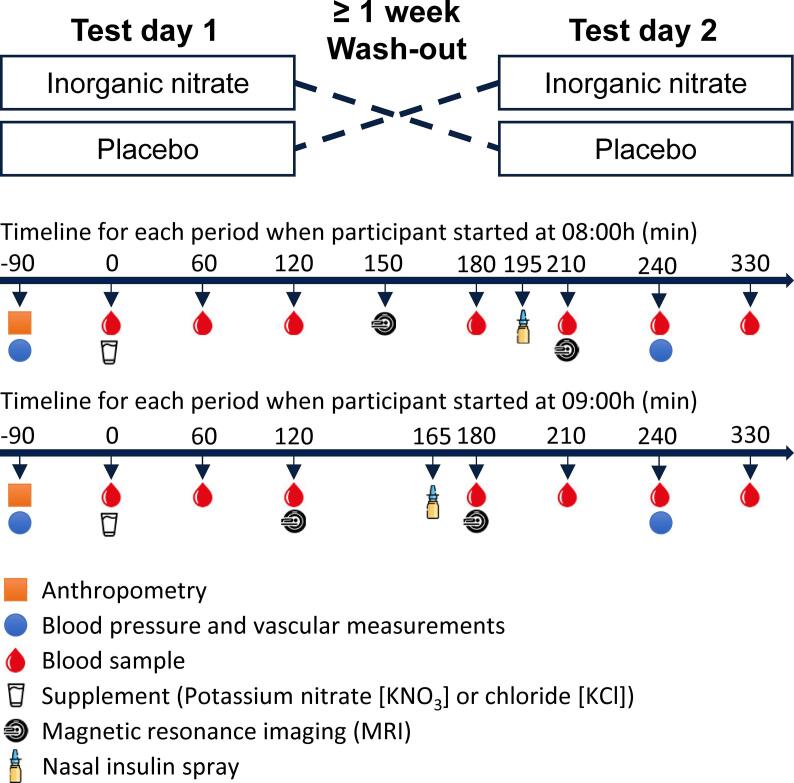
Schematic overview of study design.

This study was a randomized, double-blind, placebo-controlled, cross-over trial with a wash-out period of at least one week (median: 9 days, range: 7 – 18 days). The study design is shown in Fig. 1. Randomization was performed using software (https://www.randomizer.org/) and was concealed by the research assistant that also prepared the drinks. Participants received a drink (30 g of tap water) in which 10 mmol (i.e., 625 mg nitrate) potassium nitrate (KNO_3_; Merck KGaA, Darmstadt, Germany) was dissolved or an isomolar placebo drink with potassium chloride (KCl; Merck KGaA, Darmstadt, Germany). This dose of potassium nitrate was chosen based on the maximal reference dose for chronic oral exposure set by the United States Environmental Protection Agency (EPA; 7.0 mg per kg body weight per day, which equals 630 mg for a 90 kg individual) (United States Environemntal Protection Agency, 2020) and because it has beneficial effects on the peripheral vasculature (Lara et al., 2016, Siervo et al., 2013). For logistic reasons, participants started both test days consistently at either 08:00 or 09:00 in the morning. MRI measurements were performed 120 min or 150 min after supplementation depending on the start time at the Scannexus research facilities in Maastricht. In fact, earlier studies have clearly shown that plasma nitrate plus nitrite concentrations (Wylie et al., 2013) and the NO pool (Joris and Mensink, 2013) reached peak levels and plateaued 120 min after consuming inorganic nitrate. The insulin spray was administered between two MRI sessions and 30 min before CBF was measured for the second time, which is a common approach (Kullmann et al., 2018). Additionally, office blood pressure was measured at baseline and 240 min after the drink in supine position using an intermittent blood pressure device (Omron M7 Intelli IT, Cemex Medische Techniek, Nieuwegein, The Netherlands) (Muntner et al., 2019). Blood samples were taken from an intravenous catheter before the drink was provided (T = 0) and 60, 120, 180, 210, 240 and 330 min after the drink.

One week prior to the test day and throughout the study, participants were not allowed to use antibacterial mouth wash or antibacterial toothpaste, chewing-gum or tongue-scraping. Additionally, participants had to avoid nitrate-rich food products for which a list was provided, have a regular dinner, and were not allowed to drink alcohol on the day preceding the test day. Participants arrived by car or public transport after an overnight fast (no food or drink after 08:00 PM, except for water) at the Metabolic Research Unit Maastricht (MRUM), which is temperature controlled at 22 °C. A wheelchair was used for the transport of the participants to the Scannexus research facilities for the brain measurements. During the whole study period, study participants were kindly requested to maintain their habitual diet and use of alcoholic beverages.

### 2.3 Magnetic resonance imaging

#### 2.3.1 Acquisition

Brain perfusion measurements were performed in supine-position prior to and 30 min after intranasal insulin administration using a 3T MAGNETOM Prisma Fit MRI-system and a 64-channel head-neck coil (Siemens Healthcare, Erlangen, Germany). Insulin was administered intranasally by four puffs of 0.4 mL (two per nostril) at 30-second intervals, amounting to a total dose of 1.6 mL insulin (160 U Insulin Novorapid; Novo Nordisk, Mainz, Germany). CBF was measured using pseudo-continuous arterial spin labeling (PCASL) after 15 min of rest inside the MRI-scanner, while looking at a cross to standardize measurements as much as possible and to reduce involuntary movements. The acquisition and processing has been described in detail before (Kleinloog et al., 2019). In brief, the scan took 10 min and was performed with background-suppressed segmented three-dimensional (3D) gradient and spin echo (GRASE) readouts. The default repetition time (TR) was 4050 ms but required prolongation up to 5470 ms for some of the participants depending on the specific absorption rate (SAR) estimation. The TR was kept constant for each participant across measurements. The other sequence parameters were: TE 13.6 ms, GRAPPA 2, labeling duration 1750 ms, post-labeling delay 2000 ms, segmentation factor 6, 10 label-control repetitions with nineteen slices and a voxel resolution of 3.0 mm isotropic. Preceding each PCASL measurement one high-resolution anatomical 3D magnetization-prepared rapid acquisition with gradient echo (MPRAGE) scan (TR 2400 ms, TE 2.18 ms, TI 1040 ms, 1.0 mm isotropic resolution, 8°flip angle and 160 sagittal slices) was performed.

#### 2.3.2 Pre-processing

PCASL-images were analyzed using FSL (Version 6.0) and the BASIL toolbox (Version 4.0.15) (Chappell et al., 2009, Manjón and Coupé, 2016, Woolrich et al., 2009). First, individual PCASL-images were distortion corrected with TopUp using M0 images with opposite phase-encoding direction and a TR of 20 s. Quantification of CBF was performed following the recommendation in the ASL white Paper (Alsop et al., 2015) and assuming a labeling efficiency of 0.64 (four background suppression pulses; 0.934), a T1 of gray matter of 1330 ms, and a bolus arrival time of 1300 ms. the T1 of blood was estimated using the hemoglobin concentration of the participant measured on the test day (Li et al., 2017). Mean CBF was determined in the following pre-defined regions: global, gray matter, cortical and subcortical (i.e., caudate, putamen, thalamus, globus pallidus, hippocampus, amygdala and nucleus accumbens) after Boundary-Based co-registration to the anatomical MPRAGE image, which was segmented using Volbrain (Manjón and Coupé, 2016).

#### 2.3.3 Voxel-wise analysis

Voxel-wise comparison was performed after non-linear followed by linear co-registration to the Montreal Neurological Institute (MNI; 2 mm) using a repeated measures mixed effects analysis with a general linear model with a single-group paired difference. The effect of inorganic nitrate on brain insulin action was assessed using the difference between the post-insulin and pre-insulin CBF-maps during the nitrate and placebo test day. For evaluation of the effect of inorganic nitrate on CBF the scans after the nitrate and placebo pre-insulin administration were compared, while the effect of insulin on CBF was determined using the post-insulin and pre-insulin scans during the placebo test day. FLAME stage 1 and 2 was run. Cluster-wise interference was performed on the whole-brain excluding the cerebellum, because of issues with co-registration to the common space. We used a Z-threshold of 2.1, a voxel connectivity of 26 (P < 0.05) and included family-wise error correction based on smoothness estimates. Atlasquery was used to determine the location of significant clusters in the Harvard-Oxford (sub)cortical structural atlas.

### 2.4 Cardiometabolic risk markers

Blood pressure was measured on the left (non-dominant) arm in supine position after at least 15 min of rest in a quiet and darkened temperature-controlled room following the latest recommendations (Muntner et al., 2019). Blood pressure was measured four times, while the average of the last three measurement was used for statistical analyses.

Serum was obtained from vacutainer SST™ II Advance tubes (Becton, Dickson and Company, Franklin Lanes, New-York, USA), which were allowed to clot for at least 30 min and centrifuged at 1300× g for 10 min at 21 °C. Serum samples were analyzed for concentrations of nitrate plus nitrite (colorimetric assay; Merck KGaA, Darmstadt, Germany) at T = 0, 60, 120 and 240 min. Additionally, at baseline total cholesterol (TCH: CHOD-PAP method; Roche Diagnostics, Mannheim, Germany), high-density lipoprotein (HDL)-cholesterol (precipitation method; Roche Diagnostics, Mannheim, Germany), triacylglycerol corrected for free glycerol (TAG: GPO Trinder; Sigma-Aldrich Corporation, St. Louis, Mo, USA), and high-sensitivity C-reactive protein (hsCRP) (immunoturbidimetric assay, Horiba ABX, Montpellier) were determined. Low-density lipoprotein (LDL)-cholesterol concentrations were also calculated using the Friedewald formula (Friedewald et al., 1972). Insulin concentrations were determined in serum samples from all timepoints (ELISA, Christal Chem, Elk Grove Village, IL, USA). Plasma glucose concentrations were also determined at all timepoints using NaF-EDTA-containing vacutainers tubes (Becton, Dickson and Company, Franklin Lanes, New-York, USA), which were placed on ice immediately after sampling and centrifuged within 30 min at 1300× g for 15 min at 4 °C. All samples were immediately portioned into aliquots, frozen in liquid nitrogen, and stored at −80 °C until analysis at the end of the study.

### 2.5 Statistical analyses

Based on our previous studies (Kleinloog et al., 2019), it was estimated before the start of the study, that eighteen participants would be needed to detect a 1-SD unit change in brain insulin action between treatments with 80% power and a two-sided alpha of 0.05. This change in CBF corresponds to a change of approximately 10 to 15%, which can be expected following dietary interventions (Joris et al., 2018, Kleinloog et al., 2019) and is clinically relevant (Birdsill et al., 2013).

The statistical approach used for the voxel-wise analyses has been described under the MRI-section. All other analyses were performed using SPSS (IBM Corp., IBM SPSS Statistics, V26, Armonk, NY, USA). A two-tailed p-value < 0.05 was considered to be statistically significant. Results were first checked for normality using the Shapiro-Wilk test and are shown as means ± SDs. Only hsCRP was analyzed using the Wilcoxon signed rank test because of a non-normal distribution and results are presented as medians (interquartile range). Relations between age and insulin resistance (assessed in the fasted state using the average value of the two test days of the homeostasis model assessment of insulin resistance [HOMA-IR] (Matthews et al., 1985)) with changes in CBF were investigated using Pearson’s correlation coefficients. To test for differences in anthropometrics and the fasting lipid profile before administration of the drink, analysis of variance (ANOVA) was performed with treatment and order as fixed factors, and participant as random factor. Linear mixed models were performed to test for differences in overall brain insulin action between treatments using order, insulin spray, treatment, and insulin spray * treatment as fixed factors. The interaction term was omitted from the model, if it was not statistically significant, which made it possible to investigate the effects of treatment and the insulin spray. Participant was included as random factor and a random intercept was used. The best model fit was obtained with a Toeplitz covariance structure based on the chi-square statistic with log-likelihood values (P < 0.05), and Akaike information criterion (AIC).

The change in nitrate plus nitrite concentrates from baseline was investigated using a linear mixed model. Order, time, treatment, and time * treatment were used as fixed factors, and participant and intercept were included as a random factor. If the interaction term was statistically significant, the same timepoint of the two test days were compared pairwise using post-hoc tests with Bonferroni correction. Differences in insulin and glucose concentrations were investigated using the same model, but relative to the nasal insulin spray. The three blood samples prior and two blood samples after the insulin spray were used, because the interval between blood samples was similar. The change from the first blood sample was used as the dependent variable. Finally, linear mixed models were also used to test for differences in blood pressure using the change in blood pressure from T = 0 as dependent variable. Order and treatment were used as fixed factor and participant and intercept were included as random factor. Baseline differences in insulin and glucose concentrations, and blood pressure were investigated using a repeated measures ANOVA with treatment as a fixed factor.

## 3 Results

### 3.1 Study participants

A CONSORT flow diagram of participants throughout the study is shown in Supplementary Fig. 1. In total, 23 men were assessed for eligibility. One man was excluded because his fasting total cholesterol exceeded 8.0 mmol/L and another man because of MRI safety issues (i.e., metal implant in the jaw). Therefore, 21 men started the study. Two participants dropped out due to personal reasons before the first test day and one participant discontinued the study after the first test day because he suffered from vasovagal responses during the blood draws. A total of eighteen participants thus completed the study and were included in the statistical analyses. Participants who completed the study had a median age of 50 years (range 23 – 60 years). Their body weight (inorganic nitrate: 111.0 ± 14.8 kg vs. placebo: 111.0 ± 15.4 kg), BMI (inorganic nitrate: 33.5 ± 5.0 kg/m^2^ vs. placebo: 33.4 ± 5.0 kg/m^2^), waist-circumference (inorganic nitrate: 118.7 ± 10.3 cm vs. placebo: 118.5 ± 10.1 cm) and waist-to-hip ratio (inorganic nitrate: 1.00 ± 0.04 vs. placebo: 1.00 ± 0.04) were comparable between both test days (Supplementary Table 1).

**Fig. 2:**
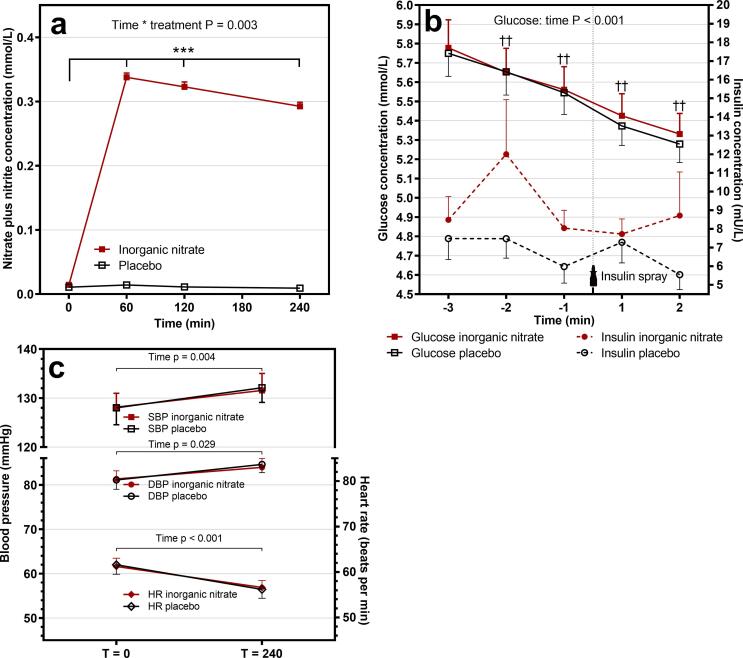
Acute effects of inorganic nitrate in a randomized, placebo-controlled, cross-over trial with abdominally obese men (n = 18). Linear mixed models with Toeplitz covariance structure was performed with time, treatment, order and time * treatment as fixed factors. Participant was included as random factor and a random intercept was used. The interaction term was omitted from the model, if it was not statistically significant, which made it possible to investigate the effects of treatment and the insulin spray. Multiple comparisons were Bonferroni corrected. (**a**) There was a significant time * treatment interaction for nitrate plus nitrite concentrations. *** After Bonferroni’s correction, nitrate plus nitrite concentrations were significantly higher at T60, T120 and T240 following the drink with inorganic nitrate (P < 0.001 at all time points). (**b**) Glucose and insulin concentration relative to the nasal-insulin spray. Glucose concentration decreased over time, †† significantly different from each other (P < 0.002). (**c**) Systolic (SBP) and diastolic (DBP) blood pressure and heart rate (HR) changed only over time.

As shown in Fig. 2a, there was a significant time * treatment interaction for nitrate plus nitrite concentrations (P = 0.003). After Bonferroni’s correction, nitrate plus nitrite concentrations were higher at T60, T120 at T240 following the drink with inorganic nitrate (P < 0.001 at all time points).

### 3.2 Brain insulin action and cerebral blood flow responses

Inorganic nitrate did not affect the CBF in response to nasal insulin administration as compared with placebo in pre-defined brain regions (i.e., global, gray matter, cortical and subcortical). Also, inorganic nitrate did not have an effect on CBF before administration of the spray (Table 1). The nasal insulin spray, however, increased mean subcortical CBF by 1.4 ± 1.4 mL/100 g/min (P = 0.001), but not in the other brain regions (Table 1).

**Table 1:** Brain insulin action and cerebral blood flow responses after administration of a nitrate and placebo drink in a randomized, double-blind, controlled crossover study with abdominally obese men^1^.

	Inorganic nitrate						Placebo						P-value 2		
	Pre-insulin			Post-insulin			Pre-insulin			Post-insulin			Treatment * insulin 3	Treatment 4	Insulin 5
	(mL/100 g/min)			(mL/100 g/min)			(mL/100 g/min)			(mL/100 g/min)					
Global CBF	41.4	±	8.1	42.2	±	8.1	42.1	±	8.6	42.2	±	8.4	0.228	0.722	0.154
GM CBF	50.1	±	10.1	50.9	±	10.1	51.0	±	11.0	51.0	±	10.8	0.278	0.695	0.374
Cortical CBF	54.7	±	11.2	55.2	±	11.2	55.5	±	11.9	55.3	±	11.6	0.703	0.760	0.649
Subcortical CBF	34.3	±	7.5	36.2	±	7.1	35.4	±	8.2	36.2	±	8.1	0.146	0.563	< 0.001

1 Values are shown as means ± SD. n = 18. CBF: cerebral blood flow; GM: gray matter.

2 Linear mixed models with Toeplitz covariance structure were performed using order, insulin spray, treatment, and insulin spray * treatment as fixed factors. Participant was included as random factor and a random intercept was used. The interaction term was omitted from the model if it was not statistically significant.

3 Effect of inorganic nitrate on brain insulin action.

4 Difference in cerebral blood flow between the nitrate and placebo test day adjusted for insulin effect.

5 Difference in brain insulin action adjusted for the nitrate drink.

**Fig. 3:**
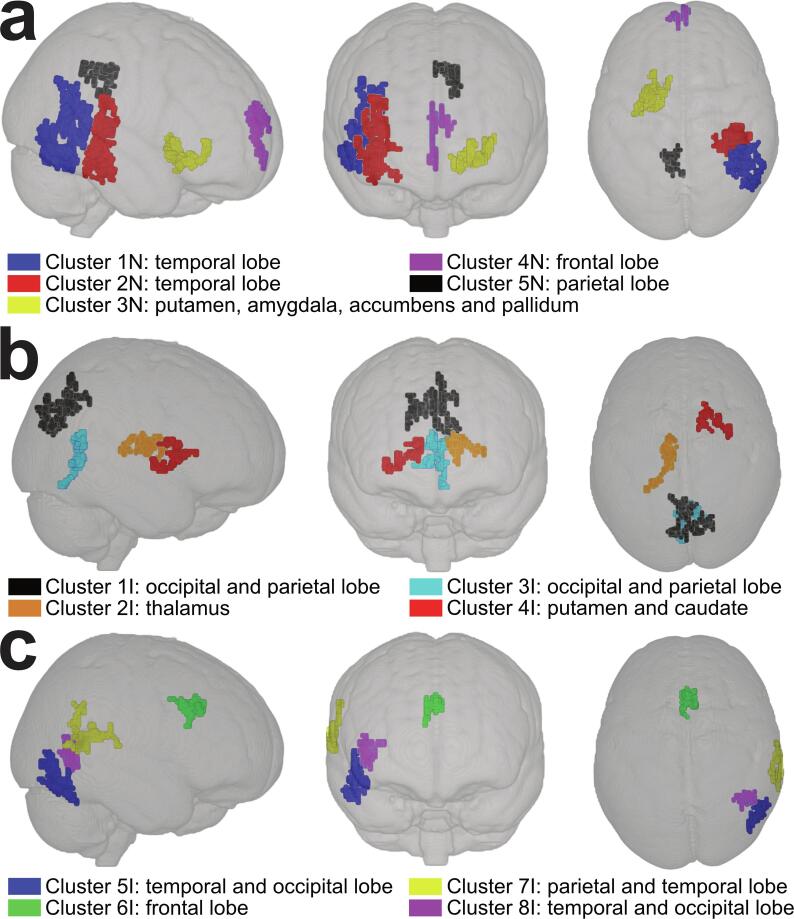
Results of voxel-wise comparisons (family-wise corrected) of the whole-brain excluding the cerebellum showing the effect of (**a**) inorganic nitrate on increased brain insulin action (treatment * insulin), and (**b**) increased and (**c**) decreased brain insulin action during placebo (insulin) in three dimensional Montreal Neurological Institute (MNI)-template from a randomized, controlled, crossover study in abdominally obese adults (n = 18). Mean changes and cluster volumes are shown in Table 1, and cluster locations in **Supplementary Table 1 and Supplementary Table 2**.

As compared with placebo, inorganic nitrate significantly increased brain insulin action in five brain clusters based on voxel-wise analyses (Fig. 3a and Table 2a). The CBF increases in response to insulin in clusters 1 N and 2 N, which were located in the right temporal lobe (36% and 26%, respectively) based on the MNI structural atlas. According to the Harvard-Oxford atlas, the specific location for cluster 1 N, was in the temporo-occipital part of the inferior (13%) and middle (7%) temporal gyrus, inferior lateral occipital cortex (6%), and temporal occipital fusiform cortex (4%). Cluster 2 N was located for 12% in the posterior temporal fusiform cortex, 3% in the parahippocampal gyrus, 3% in the inferior and 2% in the middle temporal gyrus, 3% in the planum temporale, 3% in the parietal operculum cortex, 3% in the temporal occipital fusiform cortex, and 1% in the Heschl’s gyrus. CBF in cluster 3 N increased, and was located for 23% subcortically in the left putamen (15%), amygdala (5%), accumbens (2%) and pallidum (2%), and partly in the frontal orbital cortex of the frontal lobe (3%). CBF responses to intranasal insulin following inorganic nitrate significantly increased in cluster 4 N (right frontal lobe, 61%). The specific average probability of the location was 53% in the frontal pole, 12% in superior frontal gyrus and 2% in paracingulate gyrus. Finally, CBF also increased in cluster 5 N (left parietal lobe, 58%), which was located in both the precuneus cortex (36%) and posterior cingulate gyrus (22%). The location probability of these brain clusters is also shown in Supplementary Table 2. No significant differences were observed before administration of the spray between inorganic nitrate and placebo following voxel-wise analyses.

**Table 2:** The effect of inorganic nitrate on brain insulin action (**A**) and CBF response to nasal insulin during the placebo test day (**B**) in a randomized, double-blind, controlled crossover study with abdominally obese men^1^.

A		Inorganic nitrate			Placebo			Mean difference			Volume	P-value 2
		(mL/100 g/min)			(mL/100 g/min)			(mL/100 g/min)			(mm^3^)	
Treatment * insulin	Cluster 1 N	2.7	±	3.2	−4.2	±	4.9	7.0	±	3.8	5296	< 0.001
	Cluster 2 N	3.2	±	4.0	−3.3	±	4.2	6.5	±	4.3	3592	< 0.001
	Cluster 3 N	4.7	±	2.8	−1.2	±	2.6	5.9	±	3.2	1792	< 0.001
	Cluster 4 N	4.3	±	3.5	−4.7	±	5.0	9.0	±	6.0	1096	0.007
	Cluster 5 N	4.5	±	3.6	−1.6	±	3.4	6.1	±	4.3	1024	0.012


B		Pre-insulin			Post-insulin			Mean difference			Volume	P-value 2

	(mL/100 g/min)			(mL/100 g/min)			(mL/100 g/min)			(mm^3^)	

Insulin	Cluster 1I	49.7	±	10.5	53.2	±	11.1	3.5	±	2.4	1976	< 0.001
	Cluster 2I	47.1	±	9.0	51.1	±	9.0	4.0	±	3.3	1336	0.002
	Cluster 3I	50.3	±	10.9	53.3	±	11.4	3.0	±	1.9	1064	0.011
	Cluster 4I	41.8	±	6.9	45.3	±	7.0	3.6	±	2.8	992	0.018
	Cluster 5I	50.6	±	13.8	44.0	±	12.4	−6.5	±	7.4	1368	0.001
	Cluster 6I	56.5	±	14.1	51.1	±	14.6	−5.4	±	4.3	952	0.023
	Cluster 7I	52.5	±	16.8	45.9	±	15.9	−6.6	±	5.9	944	0.025
	Cluster 8I	40.7	±	9.4	35.1	±	7.6	−5.6	±	5.1	872	0.042

1 Values are shown as means ± SD. n = 18.

2 Clusters were the result of a voxel-wise analysis applying a repeated measures mixed effects analysis using a general linear model with a single-group paired difference (FLAME stage 1 and 2), and a Z-threshold of 2.3 (P < 0.05). Family-wise error correction was performed based on smoothness estimates. The effect of inorganic nitrate on brain insulin action (treatment * insulin) was assessed using the difference between the post-insulin and pre-insulin CBF-maps during the nitrate and placebo test day (cluster N). For evaluation of brain insulin action, the post-insulin and pre-insulin scans during the placebo test day were compared (Cluster I). Inorganic nitrate did not affect CBF when the scans after the nitrate and placebo pre-insulin administration were compared.

Effects on nasal insulin spray on CBF were evaluated during the placebo test day. Insulin increased CBF in four clusters (cluster 1I – 4I; Fig. 3b and Table 2b), while a decreased CBF was found in four other brain clusters (cluster 5I – 8I; Fig. 3c and Table 2b). CBF increased in cluster 1I and 2I, which were both located in the bilateral occipital (31% and 30%, respectively) and parietal lobe (20% and 18%, respectively). CBF increased in cluster 3I, which was located in the left thalamus (79%). Finally, an increased CBF was also observed in cluster 4I (volume: 992 mm^3^) that was located in the right putamen (26%) and caudate (17%). In contrast, CBF decreased by in cluster 5I that was located in the right temporal (60%) and occipital lobe (14%). Also, a decreased CBF following spray was observed in cluster 6I (bilateral frontal lobe, 65%). In addition, CBF in cluster 7I decreased and was located in the right parietal (37%) and temporal lobe (27%). Finally, CBF decreased in cluster 8I (right temporal lobe, 29%; and occipital lobe, 10%). The specific location probability of these brain clusters is also presented in Supplementary Table 3.

No significant correlations were observed between age and HOMA-IR with changes in CBF (see Supplementary Table 4).

### 3.3 Cardiometabolic risk markers

No differences were observed in fasting TCH, HDL, LDL, TAG and hsCRP concentrations between both test days (Supplementary Table 1). No effects were observed of inorganic nitrate on serum insulin concentrations over time (time * treatment: P = 0.478). Also, no treatment (P = 0.388) or time (P = 0.100) effects were found as shown in Fig. 2b. There was no significant time * treatment interaction (P = 0.845) or treatment effect (P = 0.916) for glucose concentration, but the effect of time was significant (P < 0.001, Fig. 2b). Inorganic nitrate did not affect SBP (P = 0.764) and DBP (P = 0.538), and heart rate (HR: P = 0.346). However, SBP and DBP increased over time by 4 ± 5 mmHg (P = 0.009) and 3 ± 3 mmHg (P = 0.001), respectively, while HR decreased by 5 beats per min (P < 0.001; Fig. 2c). Baseline differences were not observed for these risk markers (insulin: P = 0.404, glucose: P = 0.680, SBP: P = 0.929, DBP: P = 0.881, and HR: P = 0.683).

## 4 Discussion

In this double-blind, randomized, controlled, cross-over trial with abdominally obese men, inorganic nitrate acutely increased the CBF response to nasal insulin in five brain clusters, which reflects an improved regional insulin action in the brain. The two largest clusters were located in the right temporal lobe (i.e., temporal gyrus and fusiform cortex), while two other cortical clusters were part of the right frontal (i.e., prefrontal) and the left parietal lobe (i.e., precuneus cortex and posterior cingulate gyrus). One subcortical cluster was located in the striatum (i.e., putamen, amygdala, accumbens and pallidum).

All cortical brain regions that showed increased brain insulin responsiveness following inorganic nitrate intake belonged to the default mode network (DMN), which comprises the lateral regions in the temporal lobe, the prefrontal and precuneus cortex, and the posterior cingulate gyrus. The DMN is a network of interacting brain regions that accounts for 90% of the energy consumed by the brain (Raichle and Snyder, 2007), which is mainly active at rest and essential for main cognitive functions such as memory and executive function (Mohan et al., 2016). The increased CBF response to intranasal insulin spray may increase the delivery of energy substrates to the DMN. This can be relevant as (glucose) hypometabolism in the DMN has been reported in patients with neurodegenerative diseases using fluorodeoxyglucose amyloid positron emission tomography (FDG-PET) (Cohen and Klunk, 2014, Mohan et al., 2016). Further, brain insulin resistance is associated with impaired cognitive performance, possibly due to changes in the connectivity of brain networks (Su et al., 2017). Therefore, the increase in brain insulin action may also apply to patients suffering from type 2 diabetes or neurodegenerative diseases like dementia who have a decreased insulin responsiveness and functional connectivity of the DMN (Albanese et al., 2017, Kullmann et al., 2016, Mohan et al., 2016). However, long-term studies in different patient groups, using different doses of inorganic nitrate, and with functional outcomes are needed to prove or disapprove this hypothesis.

The cortical ventral and dorsal striatal circuit, which consists of prefrontal, temporal and striatal clusters, are involved in the regulation of food intake by modifying specific brain reward processes (Kullmann et al., 2016). These circuits also showed an increased brain insulin responsiveness after the intake of inorganic nitrate. Both brain circuits are activated by the neurotransmitter dopamine, while insulin inhibits the action of dopamine and has anorexigenic properties by reducing the activation of these reward circuits (Figlewicz and Sipols, 2010, Kullmann et al., 2021). Interestingly, increased CBF responses to intranasal insulin in the striatum have already been observed in healthy participants (Kullmann et al., 2021, Schilling et al., 2014). In our study, similar effects were only found following nitrate intake, which may suggest that in abdominally obese participants an increased NO bioavailability is required to observe these findings. Glucose metabolism measured with FDG-PET increased both in the prefrontal cortex and in the striatum during a hyperinsulinemic-euglycemic clamp in insulin-sensitive men, while a less pronounced response was observed in insulin-resistant counterparts (Anthony et al., 2006). Similar findings were observed in response to glucose intake (Heni et al., 2014) and intranasal insulin (Kullmann et al., 2015) in normal-weight as opposed to obese individuals. It is therefore of interest if these acute effects of inorganic nitrate are also evident at the long-term, thereby possibly counteracting the reduced inhibitory control contributing to overeating behavior as has been observed in some insulin-resistant and obese individuals (Kullmann et al., 2016, Kullmann et al., 2020a).

Inorganic nitrate did not affect CBF before administration of the spray. In contrast, a study involving older adults observed an increased CBF in the frontal lobe as measured with ASL after the consumption of a high-nitrate diet, including 500 mL of beetroot juice providing 773 mg of dietary nitrate, over a 24-hour period (Presley et al., 2011). The higher dose of nitrate supplied by wholefoods over a longer period of time may explain these apparent inconsistent results. An acute increase in CBF was also observed in young adults after the consumption of beetroot juice as measured with transcranial doppler during exercise (Bond et al., 2013) or near-infrared spectroscopy during cognitive tasks (Wightman et al., 2015). These results indicate that the intake of inorganic nitrate may affect CBF following stimuli challenging the regulation of blood flow in the brain.

Abdominally obese men are known to have an impaired brain insulin responsiveness (Kullmann et al., 2020b), which is in line with the observed regional CBF responses in our population during the placebo test day. We observed an increased thalamic CBF response to insulin in abdominally obese men, which is opposite to responses following the intake of foods in normal-weight adults (Matsuda et al., 1999). Increased thalamic insulin responses were positively associated with the amount of visceral adipose tissue (Kullmann et al., 2015, Kullmann et al., 2020b). Additionally, an increased CBF was observed in substructures of the striatum (i.e., putamen and caudate), while CBF decreased in the frontal lobe (i.e., anterior cingulate gyrus). Comparable responses in these brain regions, which are involved in energy homeostasis, attention, reward, sensory perception and motivation, have previously been associated with impaired insulin responses after an oral glucose load (Kullmann et al., 2016, Page et al., 2013). In our study population, as well as in normal-weight participants (Guthoff et al., 2010), CBF was reduced in response to nasal insulin in the fusiform and temporal gyrus, and the medial part of the frontal lobe. It would therefore be of interest if the magnitude of these responses may play a role in the termination of food intake following a meal (Guthoff et al., 2010) as a more pronounced reduction in CBF in these brain regions was associated with less visceral adipose tissue (Kullmann et al., 2015, Kullmann et al., 2020b). Finally, CBF was also affected by the insulin spray in brain clusters located in the occipital lobe that are involved in the modulation of food preferences, which provides further evidence that insulin plays an important role in the regulation of processes in the brain underlying food intake and appetite (Olivo et al., 2019, Schonberg et al., 2014).

Serum insulin concentrations were not affected by the intake of inorganic nitrate and did also not change following the application of the spray. In contrast, a transient increase in serum insulin concentrations was observed 15 min after the intranasal application of 160 U of insulin (human insulin, Actrapid) in a dose-response study (40, 80 and 160 U), which was due to spillover of the spray into the peripheral circulation without affecting plasma glucose concentrations (Kullmann et al., 2018). We did not observe an increase in insulin concentrations following the application of a similar dose of insulin aspart (Novorapid). This may be due to differences between the two types of insulin in kinetics following intranasal administration of the sprays. Although we might have missed a moderate increase in serum insulin concentrations, it was however still concluded that a transient increase in peripheral insulin concentrations did not significantly affect CBF responses (Heni et al., 2017, Kullmann et al., 2018). Finally, we did observe reduced plasma glucose concentrations over time that were probably due to the long fasting period (Moebus et al., 2011), and not related to the insulin spray as glucose concentrations were also not affected in the dose-response study (Kullmann et al., 2018).

Compared with the placebo drink, the acute intake of inorganic nitrate did not affect blood pressure after 240 min, which could therefore not explain the observed differences in CBF responses in our study. In contrast, decreased blood pressure levels were observed in a recent *meta*-analysis following the acute intake of (dietary) nitrate (Jackson et al., 2018). A possible explanation might be that inorganic nitrate has more pronounced blood pressure effects when part of wholefoods (Jackson et al., 2018). In general, effects on blood pressure were only observed two-to-three hours following nitrate administration, indicating that blood pressure levels may already have been restored after four hours. An interesting observation is that blood pressure levels increased over time, while HR decreased. This could be due to the natural circadian blood pressure and HR rhythm (Shea et al., 2011). Alternatively, the insulin spray may induce sympathoexcitation of blood pressure regulatory centers in the brain, which was suggested by Benedict et al. (Benedict et al., 2005).

Our primary aim was to investigate the acute effects of inorganic nitrate on regional insulin action in the brain. Effects were tested using a whole-brain approach except for the cerebellum and involved family-wise corrections for multiple comparisons. As expected, circulating nitrate plus nitrite concentrations increased following the drink with inorganic nitrate confirming that the nitrate was well absorbed. The time from consumption of the drink to the MRI measurements differed between participants up to 30 min but was kept constant within each participant. Nitrate concentrations however remained stable during the timeframe of the measurements. Therefore, it is unlikely that this could have impacted our results. Another possible limitation is that we did not use a placebo spray (Kullmann et al., 2018) and can therefore not exclude effects due to expectation or anticipation. Also, the TR of the PCASL acquisition was as low as possible to reduce potential motion artifacts but was different between subjects due to SAR constraints. However, the TR was kept the same within participants. In addition, only abdominally obese men were included to exclude any possible sex effects (Kullmann et al., 2016, Kullmann et al., 2020b). The large age range was chosen to increase the generalizability of the results. Although it is known that CBF decreases with age (Tarumi and Zhang, 2018), no correlation was observed between age and changes in CBF. Finally, the current knowledge regarding the direction of CBF change in response to insulin and the link to functional outcomes is limited. It is therefore recommended that future longer-term studies investigate in different populations (e.g., normal-weight subjects), whether the changes in brain insulin action also translate into beneficial functional outcomes, such as improved cognitive performance and food intake regulation.

In conclusion, this study involving abdominally obese men showed that acute inorganic nitrate intake affects regional insulin action in the brain. Specifically, an increased brain insulin responsiveness was observed in regions that are involved in the regulation of various metabolic and cognitive processes in the brain, as well as in processes underlying the intake of food.

## Declaration of Competing Interest

The authors declare that they have no known competing financial interests or personal relationships that could have appeared to influence the work reported in this paper.
