# Supplementary material for: Acute inorganic nitrate intake increases regional insulin action in the brain: Results of a double-blind, randomized, controlled cross-over trial with abdominally obese men
Source: Neuroimage Clin. 2022 Jul 14;35:103115. doi: 10.1016/j.nicl.2022.103115 (PMC9421446; doi:10.1016/j.nicl.2022.103115)
Supplement: Supplementary data 1 [file mmc1.docx]

**SUPPLEMENTARY MATERIAL**





**Supplementary Figure 1.** CONSORT flow diagram of the progress through the phases of this randomized, controlled, crossover study with abdominally obese men.

| **Supplementary Table 1.** Anthropometrics and fasting lipid profile before administration of a nitrate and placebo drink in a randomized, double-blind, controlled crossover study with abdominally obese men^1^. | | | | | | | | | | |
| --- | --- | --- | --- | --- | --- | --- | --- | --- | --- | --- |
|  | **Inorganic nitrate** | | | **Placebo** | | | **Mean difference** | | | **P-value^2^** |
| Weight (kg) | 111.0 | ± | 14.8 | 111.0 | ± | 15.4 | 0.1 | ± | 1.3 | 0.857 |
| Body mass index (kg/m^2^) | 33.5 | ± | 5.0 | 33.4 | ± | 5.0 | 0.0 | ± | 0.4 | 0.759 |
| Waist circumference (cm) | 118.7 | ± | 10.3 | 118.5 | ± | 10.1 | 0.2 | ± | 2.6 | 0.752 |
| Waist-to-hip ratio | 1.00 | ± | 0.04 | 1.00 | ± | 0.04 | 0.00 | ± | 0.03 | 0.901 |
| Total cholesterol (mmol/L) | 4.86 | ± | 0.69 | 4.97 | ± | 0.87 | -0.11 | ± | 0.43 | 0.286 |
| HDL-cholesterol (mmol/L) | 1.07 | ± | 0.20 | 1.04 | ± | 0.20 | 0.03 | ± | 0.08 | 0.229 |
| LDL-cholesterol (mmol/L) | 3.17 | ± | 0.62 | 3.32 | ± | 0.62 | -0.16 | ± | 0.35 | 0.093 |
| Triacylglycerol (mmol/L) | 1.39 | ± | 0.76 | 1.33 | ± | 0.76 | 0.06 | ± | 0.30 | 0.421 |
| hsCRP (mg/L) ^3^ | 4.77 (3.85) | | | 1.20 (4.57) | | | -0.23 (0.67) | | | 0.977 |
| ^1^ Values are means ± SD. n= 18. HDL: high-density lipoprotein; LDL: low-density lipoprotein; hsCRP: high-sensitivity C-reactive protein. | | | | | | | | | | |
| ^2^ Repeated measures analysis of variance (ANOVA) with treatment and order were fixed factors, participant was added as random factor. | | | | | | | | | | |
| ^3^ Values are medians (interquartile range). Statistics were performed using Wilcoxon signed rank test due to non-normal distribution. | | | | | | | | | | |

| **Supplementary Table 2**. Location probability of the significantly changed clusters (treatment * insulin, cluster N) determined using the atlasquery function of FSL using the MNI structural atlas and Harvard-Oxford atlas. The effect of inorganic nitrate was investigated during a randomized, double-blind, placebo-controlled crossover study in abdominally obese men (n = 18). | | | | |
| --- | --- | --- | --- | --- |
|  | **Location** | | | |
|  | MNI structural atlas | Probability | Harvard-Oxford atlas | Probability |
| Cluster 1N | Right temporal lobe | 36% | Temporo-occipital part of the inferior temporal gyrus | 13% |
|  |  |  | Temporo-occipital part of the middle temporal gyrus | 7% |
|  |  |  | Inferior lateral occipital cortex | 6% |
|  |  |  | Temporal occipital fusiform cortex | 4% |
| Cluster 2N | Right temporal lobe | 26% | Posterior temporal fusiform cortex | 12% |
|  |  |  | Parahippocampal gyrus | 3% |
|  |  |  | Inferior temporal gyrus | 3% |
|  |  |  | Middle temporal gyrus | 2% |
|  |  |  | Planum temporale | 3% |
|  |  |  | Parietal operculum cortex | 3% |
|  |  |  | Temporal occipital fusiform cortex | 3% |
|  |  |  | Heschl's gyrus | 1% |
| Cluster 3N | Left subcortical |  | Putamen | 15% |
|  |  |  | Amygdala | 5% |
|  |  |  | Accumbens | 2% |
|  |  |  | Pallidum | 2% |
| Cluster 4N | Right frontal lobe | 61% | Frontal pole | 53% |
|  |  |  | Superior frontal gyrus | 12% |
|  |  |  | Paracingulate gyrus | 2% |
| Cluster 5N | Left parietal lobe | 58% | Praecuneus cortex | 36% |
|  |  |  | Posterior cingulate gyrus | 22% |

| **Supplementary Table 3**. Location probability of the significantly changed clusters (insulin, cluster I) determined using the atlasquery function of FSL using the MNI structural atlas and Harvard-Oxford atlas. The effect of inorganic nitrate was investigated during a randomized, double-blind, placebo controlled crossover study in abdominally obese men (n = 18). | | | | |
| --- | --- | --- | --- | --- |
|  | **Location** | | | |
|  | MNI structural atlas | Probability | Harvard-Oxford atlas | Probability |
| Cluster 1I | Bilateral occipital lobe | 31% | Cuneal cortex | 23% |
|  |  |  | Superior occipital cortex | 13% |
|  |  |  | Intracalcarine cortex | 16% |
|  |  |  | Supracalcarine cortex | 6% |
|  | Bilateral parietal lobe | 20% | Praecuneus cortex | 30% |
| Cluster 2I | Left subcortical |  | Thalamus | 79% |
| Cluster 3I | Bilateral occipital lobe | 30% | Lingual gyrus | 31% |
|  |  |  | Intracalcarine cortex | 16% |
|  |  |  | Supracalcarine cortex | 6% |
|  | Bilateral parietal lobe | 18% | Praecuneus cortex | 13% |
| Cluster 4I | Right subcortical |  | Putamen | 26% |
|  |  |  | Caudate | 17% |
| Cluster 5I | Right temporal lobe | 60% | Inferior lateral occipital cortex | 33% |
|  |  |  | Temporo-ocipital part of the inferior temporal gyrus | 27% |
|  |  |  | Temporo-ocipital part of the middle temporal gyrus | 2% |
|  | Right occipital lobe | 14% | Occipital fusiform gyrus | 2% |
|  |  |  | Temporal occipital fusiform cortex | 2% |
| Cluster 6I | Bilateral frontal lobe | 65% | Anterior cingulate gyrus | 64% |
|  |  |  | Paracingulate gyrus | 11% |
| Cluster 7I | Right parietal lobe | 37% | Posterior supramarginal gyrus | 20% |
|  |  |  | Angular gyrus | 6% |
|  | Right temporal lobe | 27% | Posterior superior temporal gyrus | 16% |
|  |  |  | Posterior middle temporal gyrus | 2% |
|  |  |  | Middle temporal gyrus | 7% |
|  |  |  | Planum temporale | 3% |
| Cluster 8I | Right temporal lobe | 29% | Temporo-ocipital part of the middle temporal gyrus | 16% |
|  |  |  | Temporo-ocipital part of the inferior temporal gyrus | 5% |
|  |  |  | Angular gyrus | 2% |
|  | Right occipital lobe | 10% | Inferior lateral occipital cortex | 4% |

| **Supplementary Table 4.** Pearson correlation coefficient between age and homeostasis model assessment of insulin resistance (HOMA-ir) with changes in cerebral blood flow (CBF). | | | | |
| --- | --- | --- | --- | --- |
|  | **Age** | | **HOMA-IR** | |
|  | **Pearson r** | **(P-value)** | **Pearson r** | **(P-value)** |
| Cluster 1N | 0.150 | (0.553) | -0.104 | (0.682) |
| Cluster 2N | 0.039 | (0.878) | 0.277 | (0.266) |
| Cluster 3N | 0.206 | (0.413) | 0.088 | (0.729) |
| Cluster 4N | 0.218 | (0.386) | -0.169 | (0.504) |
| Cluster 5N | -0.043 | (0.865) | 0.193 | (0.444) |
|  |  |  |  |  |
| Cluster 1I | 0.163 | (0.519) | -0.275 | (0.269) |
| Cluster 2I | 0.025 | (0.923) | 0.103 | (0.684) |
| Cluster 3I | 0.438 | (0.069) | -0.049 | (0.849) |
| Cluster 4I | -0.389 | (0.110) | -0.266 | (0.285) |
| Cluster 5I | -0.225 | (0.370) | -0.306 | (0.217) |
| Cluster 6I | -0.058 | (0.820) | -0.122 | (0.628) |
| Cluster 7I | -0.469 | (0.050) | -0.387 | (0.112) |
| Cluster 8I | -0.355 | (0.149) | 0.050 | (0.842) |
